# From flower to fruit: The origin of the trilocular ovary and fruit development in *Phragmipedium longifolium* (Warsz. & Rchb.f.) Rolfe (Orchidaceae: Cypripedioideae)

**DOI:** 10.1111/plb.70133

**Published:** 2025-11-09

**Authors:** J. P. S. P. Bento, F. Pinheiro, J. L. S. Mayer

**Affiliations:** ^1^ Programa de Pós‐Graduação em Biologia Vegetal, Instituto de Biologia Universidade Estadual de Campinas Campinas Brazil; ^2^ Universidade Estadual de Campinas, Instituto de Biologia, Laboratório de Anatomia Vegetal Campinas Brazil; ^3^ Universidade Estadual de Campinas, Instituto de Biologia, Laboratório de Ecologia Evolutiva e Genômica de Plantas Campinas Brazil

**Keywords:** capsule, lignification, mixed dehiscence, pericarp, septum

## Abstract

- While carpels in Orchidaceae are predominantly unilocular, trilocular carpels can also occur, mainly in branches separated during the early diversification of the family. Variation in septa structure in the trilocular ovaries of the anthetic flower of orchids suggests distinct ontogenetic processes. In general, Orchidaceae fruits are capsules dehiscent by longitudinal slits, however, the number of valves, the pericarp composition, and the form of dehiscence can vary. To date, little is known about the development of unilocular capsules in Orchidaceae, and even less is understood about the development of trilocular fruits and the potential mechanisms of seed dispersal. Our aim was, therefore, to evaluate development of the ovary of the trilocular fruit and the form of dehiscence in fruits of *Phragmipedium longifolium* (Warsz. & Rchb.f.) Rolfe, a terrestrial species with dehiscent trilocular capsules.
- Using light microscopy and scanning electron microscopy, we discovered that septum formation in ovaries occurs in the flower bud stage, after differentiation of the floral parts.
- Septum is formed mainly by the installation of an intercalary meristem at the apex of each placenta. After pollination and during fruit development, there are few structural changes in the ovary, besides lignification process of the pericarp. The rupture and lysis of cells of the dehiscence line and dehydration of the mesocarp result in fruit dehiscence.
- The ovary of *P. longifolium* therefore has post‐genital septum formation. Fruit dehiscence occurs by a mixed mechanism of cell rupture and lysis and fruit dehydration, evidenced for the first time in Orchidaceae.

## Graphical abstract

*Phragmipedium longifolium* has a trilocular ovary with a post‐genital septum, and during fruit development, lignification plays a crucial role in facilitating dehiscence and seed dispersal.

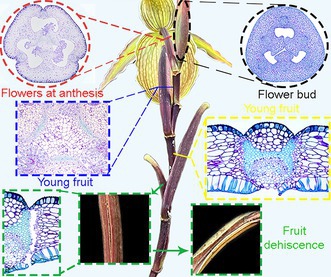

## INTRODUCTION

Unilocular ovaries are predominant in Orchidaceae, however, trilocular ovaries can also be present, mainly in early diversified clades of the Orchidaceae (Freudenstein & Rasmussen 1999; Cameron, 2004; Chase *et al*. 2015). Orchidaceae is one of the two largest angiosperm families, with approximately 736 genera and 28,000 species (Christenhusz & Byng 2016), and is widely distributed across the globe (Dressler 1993). Five subfamilies (Apostasioideae, Vanilloideae, Cypripedioideae, Orchidoideae, and Epidendroideae) are recognized based on analyses of molecular (Cameron *et al*. 1999; Cameron 2004; Kim *et al*. 2020) and morphological (Freudenstein & Rasmussen 1999) data. Four of these subfamilies have representatives with trilocular ovaries, with species in the genera *Apostasia*, *Neuwiedia* (Apostasioideae; Kocyan & Endress 2001), *Lecanorchis*, *Clematepistephium* (Vanilloideae; Garay 1960; Hallé, 1977, apud Veyret 1981), *Phragmipedium* (Cypripedioideae; Atwood 1984), and in *Palmorchis* (Epidendroideae; Veyret 1981). Besides Orchidaceae, trilocular ovaries are present in most representatives of the early diversified clades in the Asparagales (Rudall 2003), corroborating the idea that ancestral orchids exhibited a trilocular ovary (Dressler 1993).

In an extensive study of floral ontogeny in *Apostasia* and *Neuwiedia* (Apostasioideae), the origin of the trilocular carpel in Orchidaceae was described as post‐genital, occurring through the partial fusion of the carpels in the developing flower bud, with an unfused region remaining in the central ovary (Kocyan & Endress 2001). Atwood (1984) cited two studies on flowers at anthesis carried out with species of Cypripedioideae. The first study, conducted in 1854 by Reichenbach, describes the presence of trilocular ovaries in *Selenipedium*. A study by Blume *et al*. (1858) also describes trilocular ovaries in *Cypripedium* species, but unlike the ovaries in *Selenipedium*, these were trilocular at the apical and basal ends and unilocular in the median region. Thus, we still do not know exactly how the trilocular ovary develops in Cypripedioideae. Vanilloideae also lacks studies, with only a diagram/schematic drawing of the carpel of *Lecanorchis javanica* Blume (Garay 1960) and a report for *Clematepistephium smilacifolium* (Rchb.f.) N. Hallé (Hallé, 1977 *apud* Veyret 1981) published. Within Epidendroideae, *Palmorchis* species exhibit trilocular ovaries, and in *P. prospectorum* Veyret the septum contains a central lumen, and the distal portion of the ovary is unilocular, while in *P. pabstii* Veyret the septum is massive (Veyret 1981). Observations of young buds at the same developmental stage indicated a unilocular and a trilocular ovary in *P. prospectorum* and *P. pabstii*, respectively (Veyret 1981).

In Orchidaceae, fruits develop from the carpels and the hypanthium of an immature ovary after pollination (Mayer *et al*. 2011; Duarte *et al*. 2019). During this process, the ovary wall transforms into pericarp, and ovule development is resumed, so that fertilization and seed formation can occur (Mayer *et al*. 2011; Dirks‐Mulder *et al*. 2019; Duarte *et al*. 2019). Some characteristics of the ovaries, such as the number of loculi and segments/valves (Veyret 1981; Mayer *et al*. 2011; Dirks‐Mulder *et al*. 2019; Duarte *et al*. 2019), and the number of vascular bundles (Sood & Rao 1986; Sood 1989; Mayer *et al*. 2011; Duarte *et al*. 2019) persist during fruit development and are still present in the ripe fruit. Others may appear as the fruit develops, such as dehiscence lines/zones (Mayer *et al*. 2011), elaters or endocarpic trichomes (Mayer *et al*. 2011; Dirks‐Mulder *et al*. 2019; Duarte *et al*. 2019), lignified cells and regions (Sood & Rao 1986; Sood 1989; Dirks‐Mulder et al., 2019), secretory structures (Berg 1865; Havkin‐Frenkel *et al*. 2004; Mayer *et al*. 2011), and different stored substances (Alves *et al*. 2021).

Fruits protect the seeds during their development and, when ripe, are also partly responsible for seed dispersal. Most members of Orchidaceae have abiotic seed dispersal, that is, small, light seeds are carried by the wind (Karremans *et al*. 2023). Anemochory can be facilitated by elaters and other characteristics of the fruit (Cribb 1999; Mayer *et al*. 2011). Early diversifying branches in Orchidaceae exhibit a large variety of dispersal mechanisms/syndromes. For instance, Apostasioideae present zoochoric and anemochoric dispersal (Karremans *et al*. 2023). Thus, understanding the characteristics of the fruit and, if present, dehiscence can inform the diversification of seed dispersal and its different forms.

The mechanism of dehiscence in terrestrial orchids has been little explored (Sood & Rao 1988; Dirks‐Mulder *et al*. 2019), even though these species present a distinct lignification pattern. Orchid fruits can open through different numbers of valves, maintaining the apex and base fused, or just the base (Mayer *et al*. 2011; Dirks‐Mulder *et al*. 2019; Pramanik *et al*. 2023). For instance, *Cypripedium cordigerum* D. Don is a unilocular Cypripedioideae, whose dehiscence occurs due to the tension exerted by the difference in cell orientation in the endocarp tissue between the fertile and sterile valves (Sood & Rao 1988). However, it is not known whether trilocular fruits exhibit the same strategy. Studies on trilocular ovaries are generally limited to the characters of the ovaries and the flower at anthesis (Garay 1960; Atwood 1984). Kocyan & Endress (2001) present the only detailed description of floral development. Among the limited number of studies on fruits in Cypripedioideae, only one was conducted on a unilocular species (Sood & Rao 1988). Furthermore, there are structural differences in the installation of the septum of trilocular representatives (Veyret 1981; Kocyan & Endress 2001), and there is also a difference in dehiscence, with some species having indehiscent fruits (Veyret 1981) and diverse dispersal syndromes among trilocular representatives (Karremans *et al*. 2023). Nevertheless, studying fruits in Orchidaceae, particularly those with trilocular ovaries, can contribute to our understanding of their evolution.

*Phragmipedium longifolium* (Warsz. & Rchb.f.) Rolfe. is a terrestrial species of the Cypripedioideae subfamily that has dehiscent trilocular capsule‐type fruits, opening by three longitudinal slits, one per loculus. Despite this species has the widest distribution within the genus (Díaz Morales & Pupulin 2021), the ontogeny of its ovary and the development of the fruit are still poorly understood. Therefore, this species may serve as a model for understanding these processes and extending insights to other species within the subfamily. The aims of our study are to describe (i) the origin and structure of the septum of trilocular ovaries, (ii) the development of the pericarp of trilocular fruits, and (iii) dehiscence in the fruits of *P. longifolium*.

## MATERIAL AND METHODS

### Plant material

**Fig. 1:**
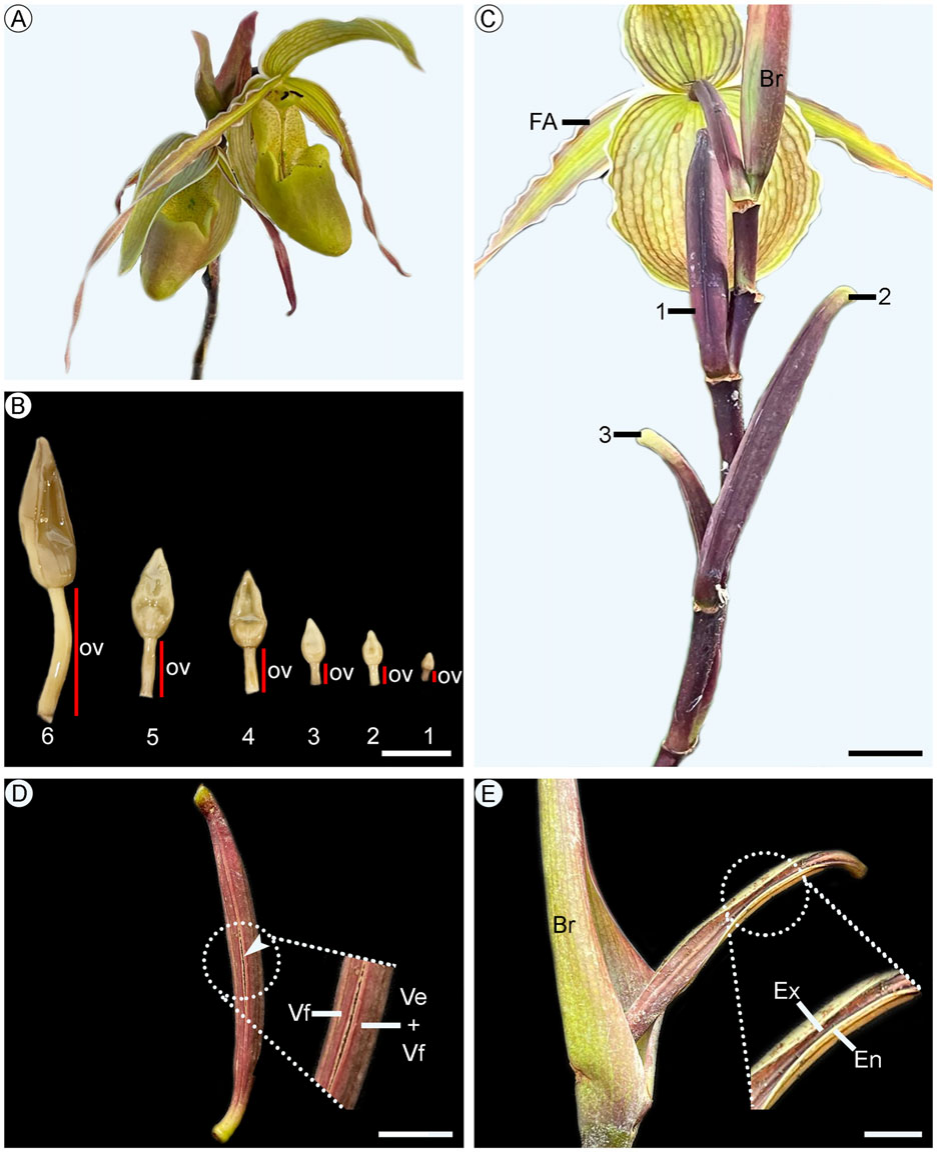
Stages of flower buds, flowers, and fruits of *Phragmipedium longifolium*. (A) Apex of an inflorescence with two flowers at anthesis. (B) Six stages of flower buds from the most immature to the most developed. (C) Three stages of fruit development from immature to the most developed. (D) Early‐stage dehiscence in the fruit, with the valves beginning to separate (indicated by arrowhead). (E) Open fruit. Symbols: Br, bract; En, endocarp; Ex, exocarp; FA, flower at anthesis; ov, ovary; Ve, sterile valve; Vf, fertile valve. Scale: 1 cm (B–E).

Three *P. longifolium* specimens (Fig. 1A), grown from seed, were obtained from a certified orchid nursery in Brazil. The specimens used in this study were in cultivation in the greenhouse of the Instituto de Biologia, Universidade Estadual de Campinas (UNICAMP), Campinas, São Paulo State, Brazil (2°49′10″ S 47°04′13″ W, 22.8195° S 47.0702° W). The testimonial materials were deposited in the form of exsiccates in the Herbarium of the Universidade Estadual de Campinas (UEC), under the number 212734.

### Ovary and fruit development of *Phragmipedium longifolium*

Ovary development was studied in inflorescence apices with flower buds at different stages of development (Fig. 1A,B). For fruit development, flowers at anthesis and fruit at different stages of development were collected (Fig. 1C–E). The fruits were collected at 7, 14, 21, 30, 60, and 90 days after pollination and after fruit opening, with at least three samples representing each stage, totaling ca. 21 samples. Based on Muñoz & Jiménez (2007, 2008), this species exhibits 100% fruit set in both manual self‐pollination and cross‐pollination. No differences were observed in morphological traits (e.g., length and width), and seed viability rates were similar between the two treatments (Muñoz & Jiménez 2007, 2008). Thus, due to asynchronous flowering among the studied individuals, fruits were produced by manual self‐pollination, without lip removal, and self‐pollination was confirmed after 3 weeks on average. Pollination is confirmed once the corolla has abscised, and the young fruit is turgid and firm. When flowers are not pollinated or when pollination is ineffective, they also lose their corolla, but the ovary becomes shrivelled until it detaches from the individual. To follow structural changes during development, the materials were observed using light microscopy and scanning electron microscopy techniques.

### Light microscopy

For the anatomical analysis, the samples collected were fixed in neutral buffered formalin (NBF; Schneider & Clark 1981), submitted to a vacuum pump to remove air contained in the tissues, gradually dehydrated in an increasing ethyl series, and embedded in synthetic resin (Leica Historesin®). Samples were then sectioned to a thickness of 5–7 μm (Pena‐Passos *et al*. 2022) using a semi‐automatic rotary microtome (Leica), stained with 0.05% toluidine blue in phosphate–citrate buffer pH 4.5 (Sakai 1973), and mounted in Entellan synthetic resin. We documented the histological slides using an Olympus BX 51 microscope with an attached Olympus DP71 video camera.

### Scanning electron microscopy

For the surface analysis, the collected materials were fixed in NBF (Schneider & Clark 1981), gradually dehydrated in ethyl series and dried using the critical point method with CO_2_ in the Balzers model CPD 030. The materials were then mounted on metal supports and coated with colloidal gold for 220 s in the Bal‐Tec model SCD 050. Analysis and electromicrographic recording were carried out using a LEO model VP 435 scanning electron microscope operated at 20 kV. All image boards were elaborated using “Photoshop CS6 Portable with 3D”.

## RESULTS

### Development of the ovary

**Fig. 2:**
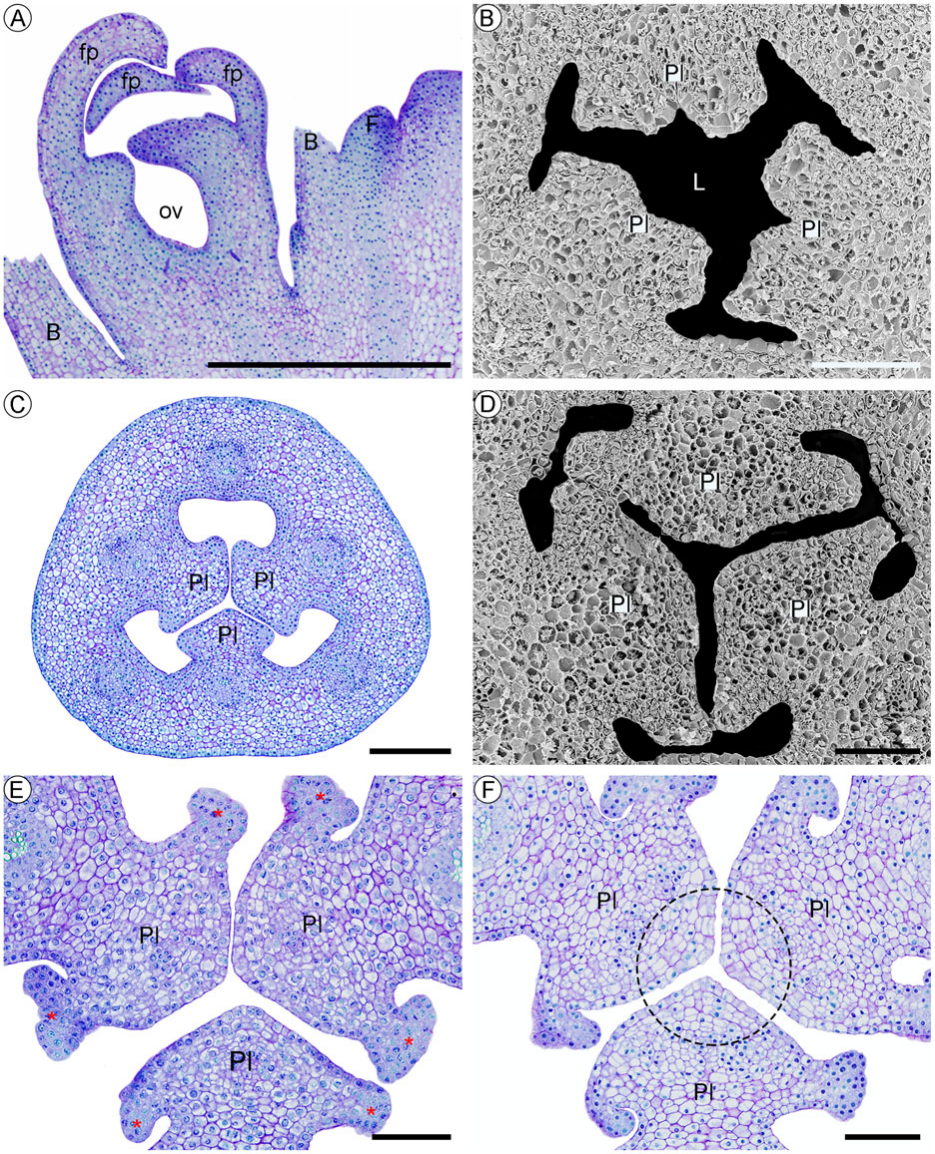
Early development of the trilocular ovary in *Phragmipedium longifolium*. (A) Apex of the inflorescence in longitudinal section, showing only one loculus in the ovary during early flower development. (B–F) Transverse sections of the median region of the ovary. (B) Early stages of the formation of the placental rudiment. (C and D) Development of the placenta, reducing the space of the loculus. (E) Early development of the egg‐bearing regions (asterisk) on the lateral part of the placentae. (F) Periclinal divisions originated at the apex of the placentae (dashed circle). Photos A, C, E, and F were acquired through a light microscope, and B and D through a scanning electron microscope. Symbols: B, bract; F, floral meristem; fp, floral piece; L, loculus; ov, ovary; Pl, placenta. Scale: 500 μm (A), 200 μm (C), 100 μm (B, D–F).

In the early stage, at the inflorescence apical portion, flower parts are barely differentiated at the apical part of the floral primordium. Subapically, there is only one loculus in the ovary, both in longitudinal (Fig. 2A) and transverse section (Stage 1 in Figs. 1B, 2B,C). In the next stage, when the bud is approximately 0.5‐cm long and floral parts are already differentiated, only one loculus and three rudimentary placental regions are visible in the ovary (Fig. 2D). With further development of the flower bud, the placental regions continue to develop and are located close to each other, and cells in the lateral ends show a clear nucleus and dense cytoplasm (Fig. 2E). In buds longer than 0.8 cm, periclinal divisions are observed in the placental region of the ovary cells. In particular, in the most median portion, and projected towards the center of the loculus, bringing the three placental projections closer together, but with only one loculus still visible in the ovary (Fig. 2F).

**Fig. 3:**
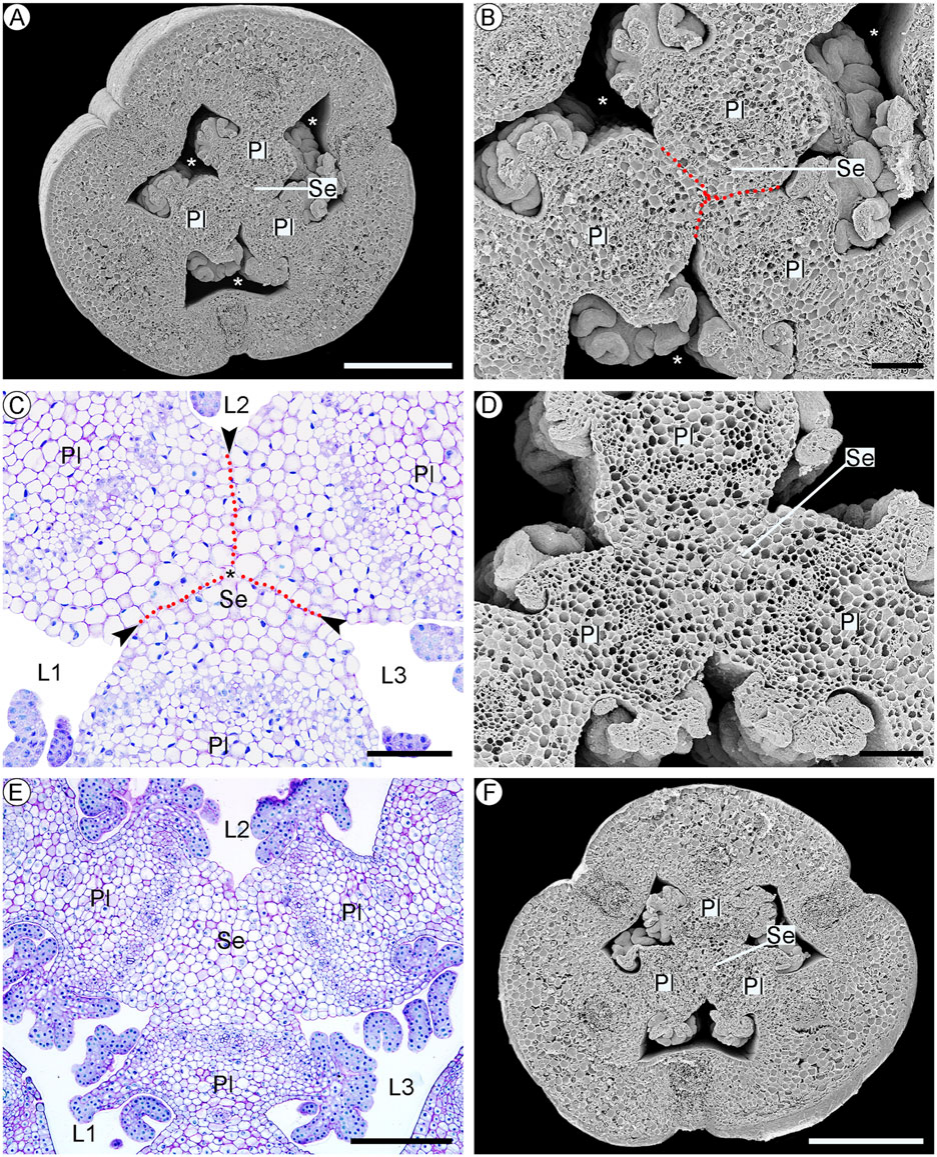
Final stages of development of the trilocular ovary of *Phragmipedium longifolium*. (A) General aspect of the recently developed trilocular ovary. (B and C) Details of the newly formed septum, indicating the areas of each placental rudiment (separated by a dashed line) and the central intercellular space (indicated by the asterisk on C). (D and E) Complete formation of the septum. (F) General appearance of the trilocular ovary in pre‐anthesis. Symbols: L, loculus; Pl, placenta; Se, septum. Scale: 500 μm (A, F), 200 μm (E), 100 μm (B–D). Photos A, B, D, and F were acquired through a scanning electron microscope, and C and E through a light microscope.

The septum of the ovary is formed as soon as the three projections of the placentae connect, related to the addition of the cells by the periclinal divisions and their cell expansion, thus forming three loculi in the ovary (Stage 4 in Figs. 1B, 3A,B), that is, a trilocular ovary. While the individual cell walls of the three placentae of the ovary are not visibly separated, it is still possible to delimit the tissue of each one because of their recent union (Fig. 3A,B). After the septum of the ovary is formed, the six margins of the three placentae are visible and will later form the ovule primordia, delimiting a central region of the septum of the ovary (Fig. 3C–F); these characteristics are preserved until the anthetic stage of the flower.

#### Fruit development

##### Ovary of the flower at anthesis

**Fig. 4:**
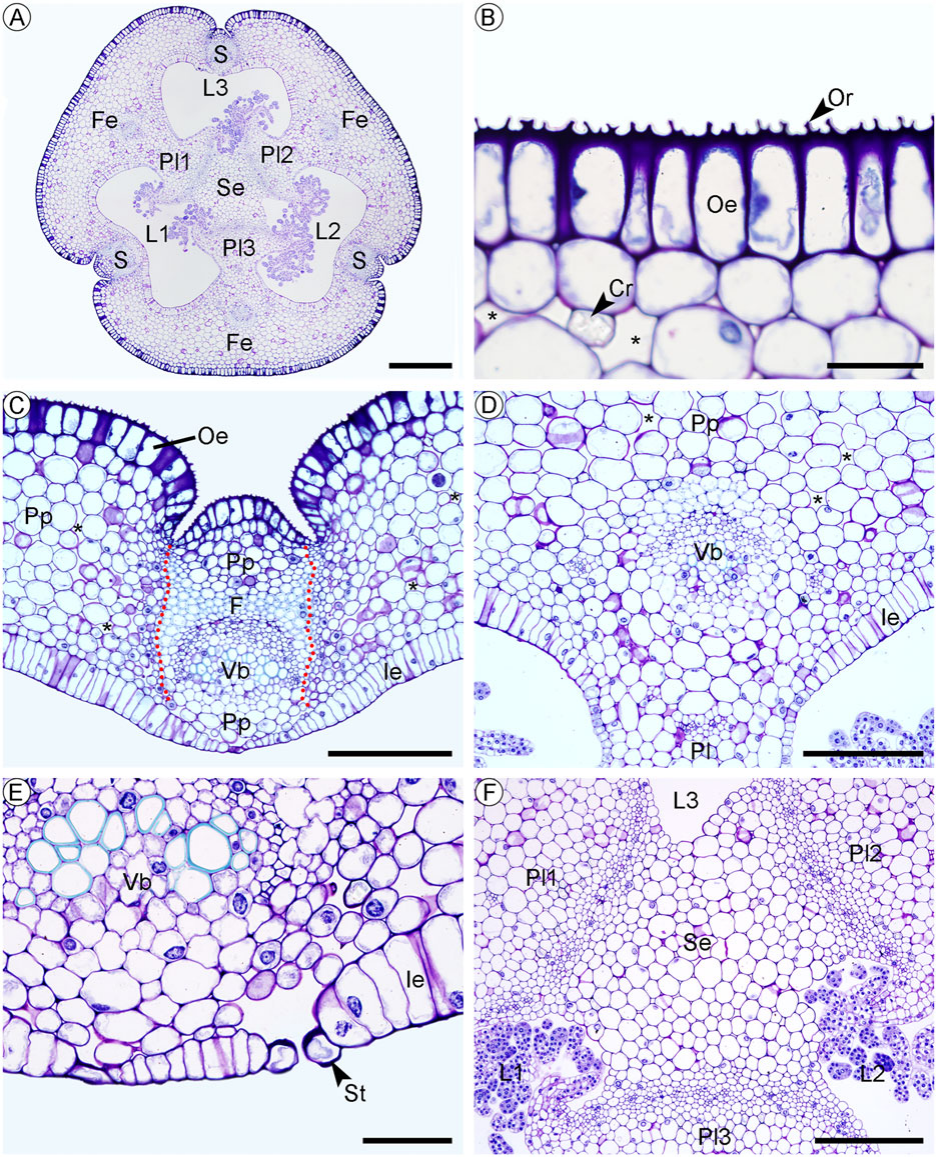
Structure of the ovary of the anthetic flower of *Phragmipedium longifolium*, in transverse section. (A) General aspect of the ovary, showing its six valves, three fertile and three sterile, three loculi, and the central septum. (B) External epidermis of the ovary, with ornamentation on the external wall. (C) Sterile valves of the ovary lack placenta, the small cells that will form the dehiscence zone are visible (indicated by the dotted line). (D) The fertile ovary valves contain the placenta. (E) Stomata occurring on the inner epidermis of the sterile valve. (F) The central septum of the fruit is formed in the region, where the three placentae meet. Symbols: Cr, crystal; F, fibres; Fe, fertile valve; Ie, inner epidermis; L, loculus; Oe, outer epidermis; Or, wall ornamentation; Pl, placenta; Pp, ground parenchyma; S, sterile valve; Se, septum; St, stomata; Vb, vascular bundle; *, intercellular space. Scale: 500 μm (A), 200 μm (C, D, F), 50 μm (B, C, E).

In cross‐section, the ovary is circular in shape, with three central loculi, six small indentations in the outer epidermis, and six distinct valves, three of them fertile (placental regions connected by the septum) and three sterile (between the placentae; Fig. 4A). The outer epidermis is uniseriate, with palisade to quadrate‐shaped cells and a thick outer periclinal wall with ornamentation (Fig. 4B). The sterile valves are smaller and lack a placenta, while the fertile valves are larger and have a placental region (Fig. 4A). Fertile and sterile valves are comprised of a dorsal vascular bundle of the carpel, bordered by fibres, with poles of ground parenchyma tissue more externally and internally to the vascular bundle (Fig. 4C). They are also delimited by areas characterized by a cluster of small cells (Fig. 4C). Fertile valves show a centralized, collateral vascular bundle opposite the placental region, which has a cap of fibres external to the phloem (Fig. 4D). Wide intercellular spaces are observed between the cells of the ground parenchyma (Fig. 4B–D), and idioblasts containing raphide‐like crystals and prismatic crystals in the ground parenchyma cells (Fig. 4B). The inner epidermis is uniseriate, with a tabular shape between the placentae and the sterile valves (Fig. 4C,D). Epidermal cells are smaller around the sterile valves, and open stomata are also observed in the region (Fig. 4E). The placentae are not fully developed and have ovule primordia (Fig. 4A, F). The septum is formed by parenchyma joining the three ends and dividing the ovary into three loculi (Fig. 4F).

##### Fruit development

**Fig. 5:**
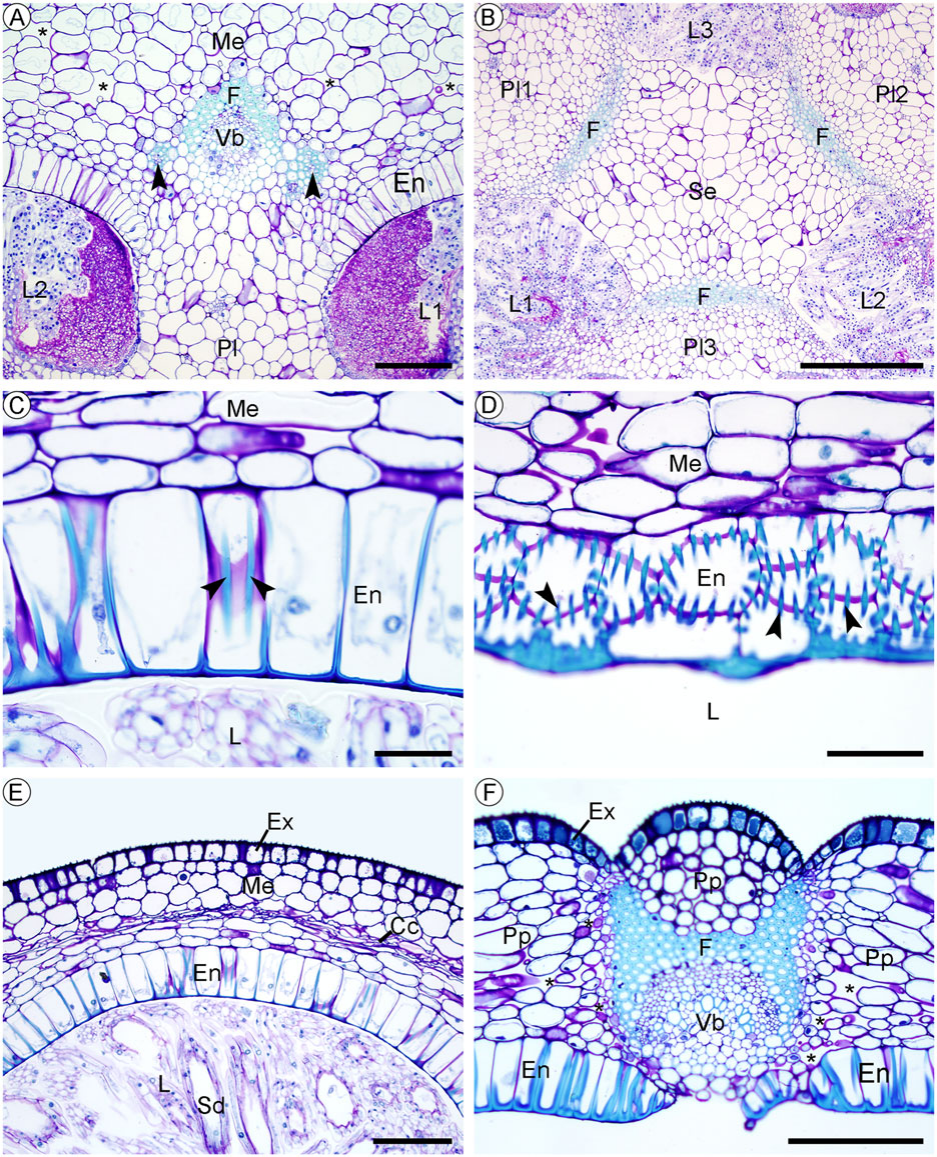
Anatomical aspects of the development of the fruit of *Phragmipedium longifolium*, in transverse section. (A) Lignification of the vascular bundle fibres, with two additional smaller vascular bundles (indicated by arrowhead) in the fertile valves. (B) Lignification of the junction zone between the placentae and the central septum. (C) Lignification of the endocarp with thickening in “beams” (indicated by arrowhead) on the side walls, giving the appearance of a U‐shaped thickening. (D) Arrowhead‐shaped lignification on the inner walls of the endocarp cells, in longitudinal section. (E) The central region of the mesocarp is composed of collapsed parenchymatous cells in the fertile valves. (F) Phenolic compounds are stored in the exocarp cells of sterile valves. Symbols: CC, collapsed cells; En, endocarp; Ex, exocarp; F, fibres; L, loculus; Me, mesocarp; Pl, placenta; Pp, ground parenchyma; Sd, seed; Se, septum; Vb, vascular bundle; *, intercellular spaces. Scale: 500 μm (B), 200 μm (A, E, F), 100 μm (D) 50 μm (C).

After manual self‐pollination and the initiation of fruit development, tissue lignification begins. This process is most evident in the fibres of the vascular bundles of the pericarp, starting in the region of the fertile valves of the smaller vascular bundles (Fig. 5A). Followed by fibres between the placental connections and the septum that isolate the central part of the septum (Fig. 5B). Finally, approximately 30 days after self‐pollination, the lignification process extends to the endocarp, with the deposition of a U‐shaped secondary wall (Fig. 5C). These depositions form “beams” on the anticlinal and the external periclinal walls, facing the fruit loculus (Fig. 5D). The mesocarp shows few changes throughout fruit development, but cells of the ground parenchyma tissue become looser with expanding intercellular spaces (Fig. 5A). Sixty days after self‐pollination, the regions of the pericarp furthest from the placenta in the fertile valves become thin due to collapse of the cells of the innermost parenchymatous cell layers (Fig. 5E), possibly related to the increased internal pressure generated by the development of the seeds. There are no visible changes in the exocarp until the end of fruit development, when phenolic compounds start to be stored inside the cells (Fig. 5F).

**Fig. 6:**
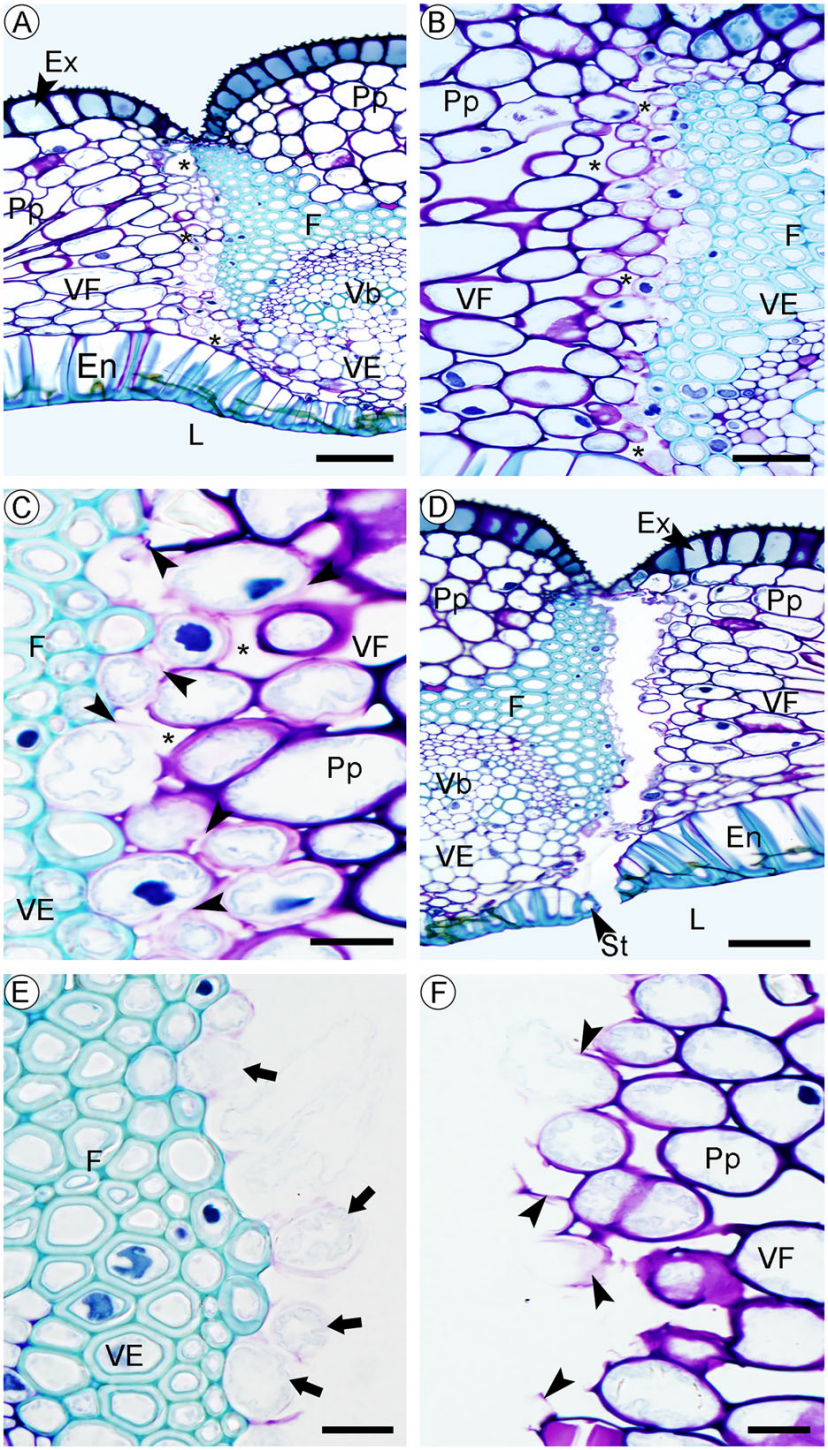
Anatomical aspects of *Phragmipedium longifolium* fruit dehiscence. (A) Dehiscence region in a mature fruit. (B) Cells in the dehiscence zone with fragile cell walls near intercellular spaces on one side and fibres on the other side. (C) The dehiscence of the fruit begins with rupture of the cell wall (arrowhead). (D) The dehiscence of the fruit first contacts the loculus through stomata. (E) Dehiscence zone, showing cells with intact cytoplasm (indicated by arrows) and remnants of degraded cell walls. (F) Dehiscence zone, showing remnants of the cell wall (indicated by arrowheads) from the cells in the dehiscence zone. Symbols: En, endocarp; Ex, exocarp; F, fibres; L, loculus; Me, mesocarp; Pp, ground parenchyma; St, stomata; Vb, vascular bundle; *, intercellular spaces. Scale: 100 μm (A, B, C) 50 μm (D), 20 μm (E, F).

Fruit dehiscence occurs approximately 92 days after self‐pollination. Fruit opening occurs through the dehiscence zone, which connects fertile and sterile valves. This connecting zone is characterized by small cells with conspicuous intercellular spaces. On the side of the sterile valve, there is a wide cap of fibres with thick, lignified cell walls, while on the side of the fertile valves, parenchymatous cells of the mesocarp are arranged transversely (Fig. 6A,B). The dehiscence of the fruit begins in the cells of the dehiscence zone located in the middle of the mesocarp, because of a combination of rupture and lysis of the thin primary cell wall of these cells, which eventually leads to the opening of the fruit (Fig. 6C). As the opening progresses and connects the edges, it reaches the loculus through the stomata in the endocarp (Fig. 6D), then it progresses to the external medium up to the innermost point of the indentations of the exocarp (Fig. 6D). After dehiscence, broken thin walls are visible in the cells, with the protoplast apparently intact (Fig. 6E). Cell wall fragments and cytoplasm of cells that have recently undergone lysis are visible at the extremity of the valves (Fig. 6F).

After the dehiscence of the fruit, the edge of a fertile valve, the adjacent sterile valve, and the edge of a second fertile valve separate (Fig. 1D). This initiates the process of seed exposure, with the edges of the valves turning outwards towards the external environment (Fig. 1E), opening through three longitudinal slits. This process lasts approximately 24 h, and after the first 12 h the fruit fully exposes 60% of the loculus cavity (Fig. 1E). The opening of the fruit and seed exposure are associated with a sequence of events, such as the rupture/dissolution of the wall of these small, thin‐walled cells and space widening already present among cells in the dehiscence zone, which allow a continuous opening from the endocarp towards the exocarp (Fig. 1D). This process exposes the parenchymatous cells of the mesocarp to dehydration (Fig. 6A–C). The parenchymatous cells near the dehiscence zone are arranged transversely (Fig. 6A, D) and, when dehydrated, they generate tension in the opposite direction to the sterile valve. This tension leads to the folding of the (fertile and sterile) valves towards the outside, similar to a window flap (Fig. 1E). The mesocarp has no lignified cells (Fig. 5E), while cells in the endocarp have differentiated lignification, keeping the inner periclinal wall with an unlignified primary wall (Fig. 5C), thus allowing the margins of the fruit to turn towards the outside of the fruit and release the tiny seeds to the wind (Figs. 1E, 5E).

## DISCUSSION

Our results, detailed through anatomical analyses, demonstrate the post‐genital formation of the trilocular ovary in *P. longifolium*, providing the first evidence of this process in Orchidaceae and confirming its occurrence in the Cypripedioideae subfamily. The lignification of the fruit pericarp is identified as a key feature for fruit dehiscence in this terrestrial orchid. Furthermore, we demonstrate that the dehiscence mechanism of *P. longifolium* fruits involves a mixed opening mechanism, which is reported here for the first time in Orchidaceae.

During early floral ontogeny, the ovaries of *P. longifolium* flower buds are unilocular, similar to representatives of Apostasioideae (Kocyan & Endress 2001), followed by formation of the septum and three loculi. Thus, the origin of the trilocular carpel is post‐genital in *P. longifolium*, similar to representatives of Apostasioideae (Kocyan & Endress 2001). However, unlike in *Apostasia* and *Neuwiedia* (Kocyan & Endress 2001), during the formation of the ovary septum the formation of a space between the placental connections in the central region was not observed in *P. longifolium*. A central space in the septum of trilocular ovaries has been described in different subfamilies of Orchidaceae, such as Apostasioideae (Kocyan & Endress 2001), Vanilloideae (Garay 1960), Cypripedioideae (Atwood 1984), and Epidendroideae (Veyret 1981), suggesting shared ontogenetic steps. However, given the evidence of two types of trilocular ovary formation in the same subfamily, which is the case of Cypripedioideae, care should be taken when analysing the ovaries of flowers already at anthesis (Reichenbach, 1854 *apud* Atwood 1984; Pfitzer, 1903 apud Atwood, Blume, 1958; Garay 1960; Atwood 1984).

Orchidaceae is a sister group to other members of Asparagales (Seberg *et al*. 2012), most species having unilocular and some trilocular carpels. Studying early diversification clades in the Asparagales, Rudall (2002) found a large variation in terms of carpel union, hypogyny, and the location of the septal nectaries. Unlike Orchidaceae, most of the early diversification families in Asparagales display trilocular ovaries (Rudall 2003). Orchidaceae do not exhibit a septal nectary, unlike most early diversifying Asparagales. However, the presence of a central space in the carpel after carpel fusion could be related to the loss of this characteristic, as observed in some members of Orchidaceae (Garay 1960; Atwood 1984; Kocyan & Endress 2001), especially in Apostasioideae, an early diversification branch (Kocyan & Endress 2001). A central nectary is observed in some Asparagales species, such as *Borya nitida* Labill. (Rudall 2002) and *Ixiolirion ledebourii* Fisch. & C.A.Mey (Rudall 2003), where the nectary is in the central position and a gap would be preserved for pollinator access.

Unlike earlier studies of orchids, we found that the trilocular ovaries of *P. longifolium* were formed by the installation of an intercalary meristem at the apex of the placenta, forming a new central tissue that makes up the septum. This process may be different or may have been ignored in Apostasioideae, due to the study technique used (Kocyan & Endress 2001). Despite the probable independent origin of trilocular ovary and its distribution in Orchidaceae (Freudenstein & Rasmussen 1999), the formation process of this character is unknown, since there are shared characteristics in its formation within the family. The trilocular ovaries may share a septum without a space or with a central space, and the ovary can be entirely or partially trilocular along its length (Veyret 1981; Atwood 1984; Kocyan & Endress 2001). For instance, *Neuwiedia veratrifolia* Blume displays the median portion of the unilocular ovary (Kocyan & Endress 2001), and *P. prospectorum* displays the unilocular distal end (Veyret 1981), while the rest of the ovary is trilocular in both species. These variations in the number of carpels within the same ovary could represent distinct ontogenetic steps in the formation of the unilocular ovaries, which are more common in the family. This raises a question about the appearance of fruits with partially trilocular ovaries. Similarly, it is unknown whether the septa of these ovaries are maintained during fruit development or if they degenerate, thus providing a single loculus with more space for the large number of seeds that are characteristic of Orchidaceae.

The ovary of *P. longifolium* can be interpreted as consisting of six portions or valves, three of which are fertile and three sterile, even though the three fertile valves are connected at the center of the fruit by the septum and the sterile valves are located between the placentae. In Orchidaceae, the most common pattern is the presence of six valves (three fertile and three sterile), originating from three carpels and only one loculus (Rasmussen & Johansen 2006), as described in the unilocular ovary of *Cypripedium cordigerum* (Sood & Rao 1988). Variation in the pattern of division of the ovary in Orchidaceae is not restricted to *P. longifolium* and other genera that exhibit trilocular, as opposed to unilocular, ovaries. The number of valves can also vary, a well‐known example is *Vanilla*, whose ovaries mostly consist of three carpels organized into two portions, reflected in its two‐valved fruits (Householder *et al*. 2010; Soto Arenas & Cribb 2010; Pansarin 2021; Bento, pers. obs.).

The fruit of *P. longifolium* is an erect, slightly curved capsule throughout its development, similar to *Acianthera johannensis* (Barb.Rodr.) Pridgeon & M.W.Chase, which are erect (Duarte *et al*. 2019), and unlike *Oncidium flexuosum* Sims., which have pendulous fruits (Mayer *et al*. 2011). However, the presence of erect fruits is a dominant character in Apostasioideae, Cypripedioideae, Vanilloideae, and Orchidoideae, but is also present in some representatives of Epidendroideae (Pramanik *et al*. 2023).

During fruit development in *P. longifolium*, there were some modest morphological changes in the fruit, mainly with regard to size and diameter, related to the increase in cell volume, similar to other representatives of Orchidaceae (Mayer *et al*. 2011; Dirks‐Mulder *et al*. 2019; Duarte *et al*. 2019; Alves *et al*. 2021). The proportion of the valves of *P. longifolium* did not change throughout development and similar to species with six valves and a single loculus (Sood & Rao 1986, 1988; Sood 1989, 1992; Mayer *et al*. 2011; Dirks‐Mulder *et al*. 2019; Duarte *et al*. 2019; Alves *et al*. 2021), the mature fruit also has three larger (fertile) valves and three smaller (sterile) valves.

The lignification process in *P. longifolium* occurs in the endocarp, in the cells surrounding the vascular bundles, and in parts of the central septum, which has not yet been described for Orchidaceae. In contrast to the lignification of the pericarp in *C. cordigerum*, another representative of Cypripedioideae, the lignification of the cell wall is restricted to the cells of the sterile valves of the fruit (Sood & Rao 1988). In other representatives of Orchidaceae, lignification involves the endocarp and also part of the mesocarp (Sood & Rao 1986; Sood 1989, 1992). In the present study, in *P. longifolium* the cells of the lignified endocarp show differential cell wall thickening in longitudinal beams, which represent a novelty for Orchidaceae. Cells with cell wall thickening in beams have already been reported as specialized cells in the aquifer parenchyma of orchid leaves (Bonfante *et al*. 2024; Moreira *et al*. 2024). Thickened cell walls increase the resilience of the cell to desiccation, preventing the cells from collapsing when dehydrated (Fradera‐Soler *et al*. 2022). However, the mesocarp of the fertile capsules of *P. longifolium* fruits is parenchymatous throughout its development, which is a more hydrated and flexible tissue, as reported for *C. cordigerum* (Sood & Rao 1988). Therefore, the thickening of the lignified cells of the endocarp, along with the lignified tissue of the septum, can play a supporting role, keeping the fruit upright and helping its dehiscence.

The dehiscence of the fruit of *P. longifolium* is initiated in the central region of the mesocarp in the median portion of the fruit, with dissolution/breakage of the wall of the small cells, which bear thin cell walls in the dehiscence zone. The separation of the cells along the dehiscence line is the first mechanism responsible for fruit opening in *P. longifolium*, a terrestrial species, differing from the process described by Dirks‐Muller et al. (2019) for an epiphytic species, in which fruit opening also involves separation of the cells of the dehiscence line. The second mechanism is mechanical, involving dehydration of the mesocarp, which is composed of parenchyma cells, and the lignified endocarp. Mechanical dehiscence had already been reported for some terrestrial representatives of Epidendroideae as the sole mechanism, driven by dehydration of endocarp cells with distinct orientations between the fertile and sterile valves (Sood & Rao 1986), which contrasts markedly with the condition in *P. longifolium*. In *P. longifolium*, as the mesocarp dehydrates, the sterile valves of the fruit open outward like windows, since the lignified endocarp is rigid and does not retract, thereby exposing the seeds to the wind. Thus, we describe a mixed mechanism of dehiscence, which has not yet been observed in any other representative of Orchidaceae. The mechanism involved in seed exposure appears to be reversible and warrants further investigation, as fruits collected and fixed in an open state returned to a closed state (pers. obs.). Field research may clarify whether this feature restricts seed dispersal following rainfall or during wetter periods.

The dehiscence process differs from the pattern reported for *C. cordigerum*, which is another member of Cypripedioideae (Sood & Rao 1988), since the orientation of the endocarp cells does not play an antagonistic role in the dehiscence of the fruit in the case of *P. longifolium*. Fruits in Oncidiinae, a tribe of Epidendroideae, seem to exhibit different forms of dehiscence. In these species, dehiscence can occur by the rupture of the small, thin‐walled cells of the dehiscence zone, as in *O. flexuosum* (Mayer *et al*. 2011), or by the formation of an electrodense layer, which covers the cells of the dehiscence zone, as in *Erycina pusilla* (L.) N. H. Williams & M. W. Chase (Dirks‐Mulder *et al*. 2019). The dehiscence of the fruits of *C. cordigerum* (Sood & Rao 1988), *O. flexuosum* (Mayer *et al*. 2011), and *E. pusilla* (Dirks‐Mulder *et al*. 2019) shows no evidence of the processes occurring at different times. On the other hand, in *P. longifolium*, there is initially a cellular action, that is, cell wall rupture and cytoplasm lysis, in the dehiscence zone that separates the valves from the fruit. This is followed by effective dehiscence through the mechanical process of dehydration, which exposes the seeds.

In the endocarp of the fruit of *P. longifolium*, cell walls are lignified and have two functions: to keep the fruit upright and to expose the seeds to the wind. The second function is possible given that the mesocarp‐facing side of the periclinal wall remains unlignified. This characteristic allows a mechanical action to occur in the parenchymatous tissue near the dehiscence zone of the dehydrated mesocarp, which pulls the margins of the fertile valves. Consequently, the valves bend outwards, exposing the seeds to the wind. The fruits of *O. flexuosum* (Mayer *et al*. 2011) and *Epipactis helleborine* (L.) Crantz (Dirks‐Mulder *et al*. 2019) similarly exhibit a lignified endocarp, as does *P. longifolium*, helping to keep the fertile valves distended. However, as observed in the pendulous fruits of *O. flexuosum* (Mayer *et al*. 2011), the fruit takes on the shape of a “Chinese lantern”, retracting the valves lengthways, thus enhancing the opening and release of the seeds. The septum of the fruit of *P. longifolium* shows lignified regions, thus maintaining the basic structure of the fruit, facilitating the escape of the seeds through the ends of the fertile valves, which are open because of dehydration of the mesocarp, similar to a window flap. Open fruits of *P. longifolium* can be closed again when immersed in an aqueous fixative after collection (pers. obs.), which we attribute to the rehydration of the mesocarp.

## CONCLUSION

In the trilocular ovaries of *P. longifolium*, the central septum is formed post‐genitally due to the installation of the intercalary meristem in the apices of the placentae. Since representatives with trilocular ovaries also occur in Vanilloideae (*Lecanorchis*, *Clematepistephium*) and in Epidendroideae (*Palmorchis*), it would be interesting to conduct ontogenetic studies to determine whether the unilocular ovary is common in these subfamilies, and whether the trilocular condition arises post‐genitally, as observed in *P. longifolium*. The fruits of *P. longifolium* exhibit mixed mechanisms of dehiscence, through cellular action followed by mechanical action. The process of lignification during the development of the fruit of *P. longifolium* is important for understanding the mechanism of seed dispersal and other aspects of reproductive biology. It is well established that wind is the main dispersal vector of Orchidaceae seeds. However, given the diversity of the family and the limited number of studies on fruit development, it is not surprising that specialized mechanisms are still being uncovered, such as that described in *P. longifolium*.

## AUTHOR CONTRIBUTIONS

JPSP Bento: Writing – original draft, methodology, investigation, conceptualization. F Pinheiro: Writing – review and editing. JLS Mayer: Review and editing, supervision, methodology, investigation, conceptualization.

## CONFLICT OF INTEREST

The authors declare no conflicts of interest.
